# Participatory Development of an International Information Brochure on the Multimodal Assessment of Disorders of Consciousness

**DOI:** 10.1111/hex.70097

**Published:** 2024-12-13

**Authors:** Melissa Hohl, Lina Willacker, Theresa Marie Raiser, Martin Justinus Rosenfelder, Katja Kuehlmeyer, Marta Bassi, Angela Comanducci, Chiara Valota, Jacobo Diego Sitt, Andreas Bender

**Affiliations:** ^1^ Institute for Medical Information Processing, Biometry, and Epidemiology – IBE, LMU Munich Munich Germany; ^2^ Chair of Public Health and Health Services Research, LMU Munich Pettenkofer School of Public Health Munich Germany; ^3^ Department of Neurology LMU University Hospital, LMU Munich Munich Germany; ^4^ Therapiezentrum Burgau, Hospital for Neurological Rehabilitation Burgau Germany; ^5^ Institute of Ethics, History and Theory of Medicine, LMU Munich Munich Germany; ^6^ Department of Biomedical and Clinical Sciences University of Milano Milan Italy; ^7^ IRCCS Fondazione Don Carlo Gnocchi ONLUS Milan Italy; ^8^ Institut du Cerveau – Paris Brain Institute – ICM Sorbonne Université, Inserm, CNRS Paris France; ^9^ Department Neurorehabilitation Medical Faculty, University of Augsburg Augsburg German

**Keywords:** disorders of consciousness, family member, health literacy, informal caregiver, information brochure, multimodal assessment, neurorehabilitation

## Abstract

**Background:**

Disorders of consciousness (DoC) refers to a group of clinical conditions of altered consciousness. To improve their diagnosis and prognosis, multimodal assessment can be of great importance. Informal caregivers of people with DoC who are confronted with new technologies as such can benefit from interventions to expand their health literacy, i.e., the ability to use information to make health decisions for oneself and others.

**Methods:**

We developed an information brochure on multimodal assessment for DoC in a participatory process, with decisions made by a steering group. The process was based on a methodological framework for the development of patient decision aids that built on the International Patient Decision Aid Standards (IPDAS).

**Results:**

On the background of a broad variety of needs, the priority was to focus on the explanation of multimodal testing and provide information about its uncertainty. Its development aimed at enhancing informal caregivers' understanding of implications of results from multimodal assessment and its relevance for prognosis. It should avoid the portrayal of information that could lead to the impression of false hope or suboptimal rehabilitation care. Informal caregivers rated its usability and acceptability highly, though they preferred less technical language.

**Conclusion:**

The participatory process was crucial to the project. Future studies should investigate the effectiveness of the brochure in fostering informal caregivers' health literacy.

**Patient or Public Contribution:**

Informal caregivers of people with DoC were deliberately included in the steering group and they participated in a field test of the prototype brochure.

**Abbreviations**
DoC: disorders of consciousness
IQR: interquartile range
M: median
MCS: minimally consciousness state
UWS: unresponsive wakefulness syndrome

## 1 Introduction

Disorders of consciousness (DoC) is a term for a group of neurological conditions which consists of clinical states following severe brain injury, such as coma, unresponsive wakefulness syndrome (UWS) and minimally conscious state (MCS). In a coma, the affected person's eyes are closed, and there is no consciousness of oneself or the surroundings [1]. When being in a UWS, eyes are open and only reflexive behaviour can be observed without any evidence of awareness of the self or the environment [2]. If a person shows behavioural signs of awareness of the self or the environment, such as command following but is incapable of communication, they are diagnosed with MCS [3].

A comprehensive diagnosis is the starting point for any health decision for people with DoC, as patients in MCS recover more frequently from DoC than patients in UWS [4]. It is of paramount importance for the prognosis, and it is the basis for decisions about therapeutic goals (e.g., regaining the ability to communicate) and decisions about treatment. Current guidelines recommend diagnosing people with DoC after a multimodal assessment in which the standardized neurological examination is combined with (functional) neuroimaging (e.g., positron emission tomography, magnetic resonance imaging) and neurophysiology (e.g., electroencephalography, nasal respiration) to improve diagnostic and prognostic accuracy [5, 6, 7, 8]. This recommendation is based on studies that demonstrate a high proportion of misdiagnoses [9, 10, 11], i.e., patients with MCS erroneously diagnosed as nonconscious. The effects of multimodal assessments are currently being investigated in the multicenter research project *PerBrain* [12].

As people with DoC are highly dependent on informal caregivers, their health literacy, i.e., the ability to use information to make health decisions for oneself and others, is important. Health literacy ‘entails people's knowledge, motivation and competences to access, understand, appraise and apply health information in order to make judgments and take decisions in everyday life concerning healthcare, disease prevention and health promotion to maintain or improve quality of life during the life course’ [13]. The concept can be related to the health of others in one's care, such as close family members facing illness or disability [14]. It becomes particularly relevant for surrogate decision‐makers. Empirical evidence suggests that high health literacy among informal caregivers can positively affect care recipients' quality of life, while low health literacy is linked to poorer health behaviours and unfavourable outcomes for the persons in care [15].

To expand their health literacy, informal caregivers require tailored information services. When people with DoC participate in diagnostic studies, their informal caregivers are confronted with especially complex and evolving information as diagnostic criteria and their examination are constantly revised and updated [7]. Providing accurate and easy‐to‐understand information can be a useful tool to help them fulfil their responsibilities and promote their own well‐being. In particular, written health information can serve as a helpful supplement during visits or consultations with doctors; it can improve health knowledge and the ability to remember the content conveyed [16, 17].

The aim of the present study was the participatory development of an information brochure for informal caregivers (family members or friends) of people with DoC who are involved in studies on multimodal assessment. The provision of background information on this new approach for diagnostic assessment was intended to support but not to replace communication between doctors and informal caregivers.

## 2 Materials and Methods

The brochure development was conducted as part of the European, multicentric research project *PerBrain* [12]. Ethical approval was provided by the institutional review board of the Medical Faculty of LMU Munich (ref. number 20‐0634).

The process was informed by a methodological framework for the development of patient decision aids [18]. We used ‘the model development process for decision aids’ based on the International Patient Decision Aid Standards (IPDAS), a literature review and pragmatic considerations [19]. The model process consists of seven phases, entailing the following steps: (1) scoping, (2) steering, (3) designing, (4) prototyping, (5) alpha testing and redesigning, (6) beta testing and (7) finalization and distribution.

As our aim was not to develop a decision aid, but rather to create a brochure that provides information on the ongoing evaluation of multimodal assessment, we adjusted the proposed process accordingly. In particular, we did not conduct a systematic literature review on the effectiveness of a measure (multimodal assessment) because the brochure was not meant to be used for decision‐making. Moreover, after testing a version of the brochure in an alpha test among informal caregivers and clinicians directly involved in the development process, the framework suggested to perform a beta test in real‐life conditions (field tests with informal caregivers and clinicians not involved in the development process) before the production of a final version. In our study, however, the beta test was only limited to a survey with informal caregivers due to limited resources. The steps were carried out as described in the subsequent sections.

### 2.1 Scoping

A core project team was built to start the working process (M.H., L.W. and T.M.R.). They involved further team members in the project planning (K.K., M.B., A.C. and A.B.). This expanded project group defined the scope and purpose of the brochure and prepared for the launch of the next phase.

### 2.2 Steering

The development of the brochure was conducted in close cooperation between the project group and a steering group, consisting of members of the *PerBrain* project (*n* = 8) as well as experts (*n* = 9) with different (professional) backgrounds. Members of the steering group were suggested by the expanded project group and had to speak English sufficiently. Two participants were deliberately selected because they were (current and former) informal caregivers of people with DoC. Furthermore, the steering group included healthcare professionals working at two neurorehabilitation facilities in Germany and Italy (two *PerBrain* consortium partners). The members represented the following disciplines: health communication, medical ethics, neurology, neurophysiology, neuropsychology, nursing, pastoral care and journalism. Experts from clinical institutions and persons working outside a clinical context were represented. Persons from France and the UK were also included. The brochure was first developed in English, and subsequently translated into German, French and Italian (three of the four languages of the *PerBrain* consortium partners).

In total, four online meetings were held with the steering group which provided advice and made decisions about the brochure during the different phases regarding both the development process and the content, layout, or dissemination strategies of the brochure. During the meetings, participants were discussing different brochure prototypes, as well as the results of the alpha and beta tests. Updates were presented by MH at the beginning of each meeting. After each meeting, the members of the core project team (M.H., L.W., T.M.R.) and further authors incorporated the jointly discussed ideas into the next draft brochure. The core team provided updated versions to the members of the steering group before the next meeting.

### 2.3 Designing

#### 2.3.1 Narrative Literature Review

Before the first steering group meeting, we conducted a literature search for two narrative reviews. A four‐step online search strategy [20] was used.

The first part of the literature search was aimed at informing a narrative review on research findings about the informational needs of informal caregivers of people with DoC [21]. Three blocks of keywords were identified and combined using the AND search command (see Table 1) [22]. Search inclusion criteria were defined as follows: (1) German or English language; (2) investigation of the needs of relatives, especially the need for medical information on DoC; and 3) experience reports of informal caregivers of people with DoC. The search strategy was used to search the Medline and PsycInfo databases on the EBSCO*host* search platform [22] (search performed on 28 February 2022). Of the initially identified 59 studies, 13 met the inclusion criteria.

**Table 1:** English search terms used for narrative review which were combined with the operator ‘AND’.

Block 1: Terms related to the clinical picture	Block 2: Terms related to information	Block 3: Terms for caregivers
Disorder of consciousness **OR** disturbance of consciousness **OR** unresponsive wakefulness syndrome **OR** vegetative state **OR** minimally conscious state **OR** traumatic brain injury	Information needs **OR** information acquisition **OR** information dissemination **OR** education expect **OR** information expect or brochure **OR** information material	Caregiver **OR** family caregiver **OR** informal caregiver

The second part of the narrative literature review was focused on grey literature. We searched for web‐based information brochures for informal caregivers of people with DoC entailing information on diagnosing, treating and prognosticating DoC. It had two functions: to specify the need for the information brochure and to provide suggestions for the possible content of the brochure.

The knowledge served as a working basis for the core team and informed the creation of the brochure for informal caregivers by guiding its content and structure. It provided arguments for decisions about the scope of the brochure, the tasks in the several phases of the brochure development process, priorities in topics and content and communication style. It particularly helped to anticipate common questions caregivers had about the DoC and neurodiagnostic measures.

#### 2.3.2 Steering Group Work

In the first steering group meeting, the research project *PerBrain* as well as the results of the narrative literature review were presented. The content of the brochure was discussed for the first time. Accordingly, an initial general topic outline for the brochure was created. The topics were structured in several chapters. The steering group members provided suggestions for text sections and were invited to co‐author the brochure together with the core team. Furthermore, the involvement of experts for easy language in the further development of the final brochure version was recommended. The core team was entrusted with the task of drafting the text.

### 2.4 Prototyping

Based on the results of the designing phase and considering recommendations for developing health information material [23, 24], M.H. and L.W. created two initial prototype brochure versions. Both of them were sent to all authors and steering group members by e‐mail and were presented and discussed in a second online meeting. The two drafts differed in terms of content and format. Options were chosen by the steering group and aspects of the prototype were discussed. All change requests were implemented into an updated prototype version by M.H., L.W. and TMR. Regarding the easy language of the English brochure prototype, advice from a freelance science journalist was included, emphasizing the importance of easy language to ensure comprehensibility to foster health literacy [25].

### 2.5 Alpha Testing and Re‐Designing

We conducted the alpha test among people directly involved in the development process by use of e‐mail correspondence. We sent the prototype out for feedback to the steering group members, including (current and former) informal caregivers and healthcare professionals in rehabilitation centres in Germany and Italy. Feedback was discussed in a third online meeting where the authors and the steering group agreed upon a structure of the final prototype (Supporting Information S2). After the meeting, the developed prototype was translated into Italian, French and German by native speakers. The three versions were subsequently checked for linguistic consistency. A plan for the beta testing was agreed on.

### 2.6 Beta Testing

To assess the usability, comprehensibility and acceptability of the brochure prototype, a beta (field) test was conducted with caregivers of people with DoC. Tests were planned for participants in Germany, Italy and France using the prototype translated into the respective country languages. Since there was no project partner in an English‐speaking country, the English prototype itself was not tested among informal caregivers. Only data from Italy and Germany were considered in the final evaluation, since there was only one participant in France, and the French clinical setting was not fully comparable to the ones in Germany and Italy.

Caregivers were recruited from two neurorehabilitation facilities (Therapiezentrum Burgau, Germany and IRCCS Fondazione Don Gnocchi, Italy). Both centres are specialized for DoC and have implemented multimodal testing, including functional neurodiagnostics, into their diagnostic procedures. Family caregivers of persons with a subacute or chronic DoC were selected from those involved in the *PerBrain* project [12] Selection for the field test followed the principles of purposive sampling and maximum variation [26]

Attention was paid to purposefully choose caregivers with a variation in the following characteristics:

o Age: Comprehensibility of the novel diagnostic technologies and usability of QR codes for elderly readers should be assessed
○ Gender: Predominantly women take on the role as caregivers [27]; however, to avoid a gender bias, both genders' perspectives should be considered
○ Relationship to the person with DoC: mother/father, sister/brother, spouse, daughter/son
○ Educational level: Lower health literacy has been linked to lower education levels [28, 29]
○ Role of informal caregiver: primary caregiver; surrogate decision‐maker (e.g., power of attorney or legal guardian)

A structured questionnaire was developed for the field test. It was divided into the same chapters and sections as the presented brochure prototype and contained questions on 6‐point Likert scales ranging from 1 (‘Strongly disagree’) to 6 (‘Strongly agree’) as well as open‐ended questions. The latter were used to evaluate each chapter and to generate specific suggestions for improvement. Furthermore, a set of scaled questions about the brochure layout and design, as well as concluding questions (e.g., ‘What do you think of the cover?’, ‘What did you think when you read the explanatory introduction about the use and meaning of the brochure?’) were included. Caregivers' responses to the open‐ended questions were translated into English by the interviewers.

The brochure prototype and the questionnaire were sent to caregivers as PDF versions via e‐mail 2–3 days before the survey took place with an invitation to read the prototype. Flexibility was granted about survey administration (in person, video call or telephone interview) under the condition that it would be conducted in a calm, distraction‐free setting. Microsoft Excel (version 2211) was used for descriptive analysis of the survey data. Results from scaled questions were expressed as median (*m*) and interquartile range (IQR), due to the small sample size. Open‐ended questions were analysed by grouping identical statements and sorting them by frequency [30]. Qualitative data were analysed using the technique of summarizing within content analysis after Mayring [30].

### 2.7 Finalization and Distribution

The results from the beta test were analysed and presented to the steering group in a fourth online meeting (M.H.). Based on the ensuing discussion, further improvements and adaptations of the English prototype were incorporated into the final brochure version (Supporting Information S2). Further recommendations for adaptations to different care contexts and personalization to individual caregivers and patients were also included.

## 3 Results

We consider the realized participatory development process as the result of the study. The brochure is an artefact of this process. It is added as Supporting Information S2.

### 3.1 Information Needs of the Target Group

The narrative review of the information needs reported by informal caregivers of people with DoC revealed that, despite challenges in understanding medical terminology, most informal caregivers wanted to be informed about DoC. They especially wanted to get information about the current condition and possible future evolution of the persons they cared for [31]. If they perceived a lack of knowledge, they worried about not being able to properly fulfil their caregiving role [32]. Specifically, information about diagnosis and prognosis as well as details about what drives medical decisions and what elements are used to determine the course of action were desired, including possibilities and limitations of certain treatment strategies [33, 34, 35]. Caregivers wished that medical professionals proactively shared such information with them, as asking physicians for information could sometimes be perceived as potentially intrusive [32]. A lack of adequate information provided by health professionals was indeed identified as one of the most frequently cited problems by caregivers of people with an acquired brain injury [36, 37]. Informal caregivers sometimes reported that they experienced only few opportunities to communicate with healthcare providers as well as a lack of recognition of their role in the process [38, 39]. They describe communication with medical staff as potentially difficult, especially when they had to initiate it themselves [40]. Furthermore, they often reported that there was not enough time to adequately address all their questions and concerns [33, 41]. Sometimes they also failed to fully grasp and understand the delivered information [42]. Thus, they frequently ended up acquiring and researching information through a web‐based search strategy or face‐to‐face conversations outside of the care setting (e.g., support groups) [16, 43]. Table 2 in Supporting Information S1 gives an overview of the retrieved information from the articles and shows how it was implemented in the brochure development.

### 3.2 Brochure Development

Through the online search for brochures on DoC we retrieved 13 publicly available German‐ and English‐language brochures dealing with the topic of severe brain injuries and their consequences provided by the following organizations: Brain Injury Association of America [44]; Bundesverband Schädel‐Hirnpatienten in Not e.V., Deutsche Wachkomagesellschaft [45]; California Pacific Regional Rehabilitation Center and the CPMC Foundation [46]; Defense and Veterans Brain Injury Center [47]; Delaware Health and Social Services, Division of Services for Aging and Adults with Physical Disabilities, Coma Task Force [48]; Fachgruppe Sozialarbeit der Landesarbeitsgemeinschaft Phase F Sachsen [49]; Freie und Hansestadt Hamburg – Behörde für Gesundheit und Verbraucherschutz [50]; ZNS‐Hannelore Kohl Stiftung [51, 52]. A variety of topics was covered within these brochures including long‐term care, rehabilitation and opportunities for financial support [44, 45, 46, 47, 48, 49, 50, 51, 52, 53, 54, 55]. No information brochure for informal caregivers specifically addressed multimodal assessment.

It was thus decided that the brochure should fill in the gap of specific information material for multimodal assessment and, at the same time, provide background knowledge of DoC, including explanations about their potential diagnosis and related uncertainty, as well as the relationship between diagnosis and prognosis. In light of the latest European and American guidelines and recommendations on the diagnosis of DoC, it was deemed important to emphasize that results from single diagnostic measures should not be interpreted in isolation [6, 7]. Rather, they ought to be considered as interrelated components that collectively contribute to a comprehensive and personalized understanding of a person with DoC diagnosis [6, 7].

It was decided to leave out information about rehabilitation measures and their potential outcomes, as this information would have to be portrayed in relation to their availability in specific countries, facilities, and their potential fit with different DoC groups. Likewise, there were other topics of great importance to informal caregivers that did not concern neurodiagnostics (e.g., legal or financial issues, psychosocial burden) and were not included in the brochure. By devising the brochure with a modular structure, these topics could potentially be expanded by country‐specific information later on. The general topic outline for the brochure included chapters on the following aspects: Definition and meaning of DoC (aetiology, diagnostic categories), currently available diagnostic measures (including their limitations), capabilities of people with DoC according to their diagnoses, implications of results from multimodal assessment (including relevance for prognosis), caregivers' expectations regarding the chances of recovery and the extent to which a recovery can be expected.

At first, there was a discussion among the steering group members about the formulation of the chapter dealing with caregivers' expectations. Disagreement was centred on how to portray functional residual cognitive and behavioural abilities of people with DoC, in order not to create false hopes of recovery among informal caregivers. Agreement was reached about the need to highlight the lingering diagnostic uncertainty, even when using multimodal assessment. It was deemed important to emphasize that the residual behavioural capabilities of persons with DoC can be perceived differently and may exhibit recurrent fluctuations over time.

As it was possible to choose between two brochure formats (A4 or A5), the preferred format was A4 to better display the amount of information, with a note to re‐evaluate the format when field testing the brochure prototype with caregivers. Chapter summaries were highlighted in colour, and pictures of diagnostic methods, a glossary, and a list of abbreviations were added. Colour‐coded interactive note boxes and text fields were included at the end of each chapter so that readers could individually note any ambiguities or questions to the health professionals. QR codes to access additional web‐based materials were provided in the French, German and Italian versions of the brochure, but not in the English language prototype. Contact information for support groups, important websites or telephone numbers were also provided for further information. Authors of the brochure, date of creation, funding information and source citations were added.

### 3.3 Beta Testing With Informal Caregivers

Beta tests of the prototype were conducted with 12 informal caregivers (Germany: *n* = 6, Italy *n* = 6; 6 females, 6 males). Participants ranged in age from 27 to 66 years (mean age: 52 ± 11.6 years). Educational level ranged from middle school to university degree (middle school: *n* = 7, high school: *n* = 3, higher education (university degree): *n* = 2). Spouses (*n* = 5), parents (*n* = 3), siblings (*n* = 2) and children (*n* = 2) of a person with a subacute or chronic DoC were represented. The role of the informal caregiver was balanced between the options of lasting power of attorney (*n* = 7) and a legal guardian appointed by the court (*n* = 5). Overall, six of the surveys took place in person (Italy), one via video call and five as a telephone interview (Germany).

Overall, the brochure was reviewed positively by participants. The order of the content was generally perceived as favourable. Ten participants indicated that the number of abbreviations throughout the brochure should be limited. Participants disagreed about the cover which only displayed the brochure's title and date of creation: six participants found it too neutral and would have preferred an embedded picture; by contrast, the other six rated it as good and stated that no picture was necessary. The introduction was perceived to be appropriate (7/12). Five participants felt well addressed in it, and four stated that it had a personal note. For Section 2.1, comprehensibility ratings revealed that median scores amounted to 5 (IQR = 2) in Germany and 3.5 (IQR = 1.75) in Italy. For Section 2.2, differences between participants in Germany and Italy were found for usefulness and comprehensibility ratings. Usefulness achieved a median score of 6 (IQR = 1.25) in Germany, and 3.5 in Italy (IQR = 2). Comprehensibility was rated with a median score of 5 in Germany (IQR = 1.25) and 3.5 in Italy (IQR = 1.25). Group differences in usefulness and comprehensibility were also detected for Section 2.3 (usefulness: *m* = 5, IQR = 1 for participants in Germany; *m* = 3.5, IQR = 2 for participants in Italy; comprehensibility: *m* = 5, IQR = 1.25 for participants in Germany; *m* = 3, IQR = 1.5 for participants in Italy). In Section Patient or Public Contribution, 5, participants in Italy rated the amount of information with a median score of 5 (IQR: 2.5) whereas participants in Germany rated it with a median of 3.5 (IQR: 3.3).

We provide a detailed report on informal caregivers' opinions about the brochure chapters in Table 3 of Supporting Infrmation S1. Additionally, an overview of the quantitative scores (median and IQR) is provided in Table 4 of Supporting Information S1. Space for notes was rated more useful by participants in Germany (*m* = 5.5, IQR = 1.75) than in Italy (*m* = 3, IQR = 3.5).

### 3.4 Finalization and Distribution

Since informal caregivers highlighted the need for further clarifications about the availability of multimodal tools in different facilities (see Table 4 in Supporting Information S1), we included the information that not all hospitals offer multimodal assessment, nor do they offer all diagnostic measures presented in the brochure. To mitigate an assumed fear of missing out, a caveat was added to clarify that the choice of clinical facilities where certain diagnostic methods are not available does not necessarily imply a lower quality of care. Additional emphasis was placed on the inherent uncertainty surrounding prognosis in general, as well as the possible ambiguity when results of multimodal assessments become available. It was noted that future research might lead to new insights which could be included in updated versions of the brochure. Boxes containing space for further information sources were not prefilled in the final English version, but it was suggested that the final translated versions should have fields containing country or hospital‐specific contact details. Abbreviations were reduced throughout the whole document and provided in a glossary. Phrases were rephrased into easy language.

It was suggested that the brochure should be offered to every informal caregiver of people with DoC who participate in multimodal assessment and that it should preferentially be distributed by medical personnel. In addition, it should be freely accessible on the internet. Ideally, the brochure should be used to support conversations with medical professionals and serve as a resource that could be taken home for discussions with friends and family. Emphasis was placed the brochure should not serve as a substitute for discussions between caregivers and medical professionals, nor should it be utilized as a tool for medical decision‐making.

## 4 Discussion

### 4.1 Discussion of the Research

Through a participatory process, a brochure addressing caregivers' information needs on the introduction of multimodal assessment to persons with DoC was developed to complement the communication of caregivers with health professionals. The initiative is innovative as it has rarely been used for the development of information material for informal caregivers of seriously ill populations [1, 2]. The development process was adapted from ‘the model development process for decision aids’ which was based on the International Patient Decision Aid Standards (IPDAS), a literature review and pragmatic considerations [19]. Although the framework was not developed to design information brochures, it was successfully adapted to the purposes of our study, and the brochure benefitted tremendously from the interdisciplinary steering group as well as from the beta testing with informal caregivers. The process did not benefit from all the steps proposed in the framework. While decision‐aids often integrate results of evidence synthesis [56], an information brochure about novel diagnostic measures which are currently under study cannot rely on similar content. Our scoping review highlighted caregivers' needs, while the recommendations derived helped to prioritize tasks and structure the process. Yet, these recommendations are not novel to experienced health professionals in neurorehabilitation and could have also been put forward by members of the steering committee.

Particularly regarding comprehensibility and usefulness, differences in the descriptive data between the participant groups in the two countries, Germany and Italy, were observed. The sample was too small to determine whether the differences were statistically significant. Potential reasons requiring further testing could be that the translated brochures differed in the use of easy language, or that the demands and preferences of the participant groups differed. With regard to readability, a study on educational material about strokes showed that the material offered required a higher educational level than the one that can be expected from stroke patients [57]. The researchers used the SMOG index, a writing tool that helps writers score their work for readability and clarity of message [58]. In a similar study, researchers comparatively assessed the readability of educational material that major US‐American cerebrovascular healthcare organizations provided through the Internet using eight different assessment methods [59]. Such tools could have been useful in the writing process and could help to further improve the development of multilingual information brochures like the one developed in our study especially to examine translations of brochures in different languages. Possible differences could further be related to participant characteristics (education), their prior knowledge of DoC or their decision‐making preferences. Systematic translations using forward–backward translations could be used to minimize the influence of translation. Such differences in preference and evaluation could be systematically assessed in future studies.

A brochure is only one means to provide educational resources to people with acquired brain injuries or their informal caregivers and might not be preferable for all types of informal caregivers. A systematic review of information provision in neuro‐oncology revealed the additional use of verbal communication (either in person, via phone or videoconferencing system), videos/films, enhanced patient admission documentation, internet‐based self‐help courses, open group sessions and educational workshops [60]. The effectiveness of these resources was not only evaluated in terms of information provision and satisfaction with the intervention but also of the gained knowledge in a pre‐/post‐design. Particularly, written health‐related information was shown to serve as a helpful supplement during consultation and advice with doctors, improving health knowledge and the ability to remember conveyed contents [16, 17] as well as allowing caregivers to grasp the meaning and consequences of their loved one's condition [61, 62, 66]. Yet, presenting information in a brochure format may not be suitable for users with native languages other than the language used in healthcare provision or with a low level of education. The development of additional information sources and tools to improve communication between informal caregivers and medical professionals and is thus desirable and could be the subject of follow‐up projects. Other sources could be communication training programs for medical staff, internet‐based self‐help courses, or face‐to‐face workshops for caregivers [60, 63]. From this perspective, the development of brochure versions adapted to specific care settings should not be limited to conveying multimodal assessment information, which is just one of the possible aspects of health literacy of informal caregivers of people with DoC. A different approach to test the brochure could be to investigate its effects on informal caregivers' psychological status when people with DoC are admitted to a rehabilitation care facility. It is known that the psychological burden of informal caregivers can be high, especially at the beginning of the rehabilitation process [64]. A study investigating the preferability of different ways to display medical information about surgery to patients before surgery found that written material was especially effective in controlling patients' anxiety [65].

On the other hand, a brochure could have unwanted side effects. Retrieving medical information about the current condition and rehabilitation potential of people with DoC can potentially add a burden to caregivers. In a qualitative study with family caregivers at a rehabilitation facility in Germany, participants reacted differently to professional evaluations of a patient's condition based on the value they attributed to the information [66]. Particularly, they considered information that had a ‘positive’ value (i.e., when doctors/researchers detected signs of conscious awareness in the patient) differently from information that had a ‘negative’ value (no detection of signs of conscious awareness). Hence, to maintain their hope of recovery, caregivers in that study adapted information selectively. Interestingly, the present study found that parts of the information provided in the brochure (e.g., data on the probability of emergence from MCS) were regarded as hard to understand as well as frustrating, potentially conveying a similar ‘negative’ value to participants.

### 4.2 Limitations and Impact on Future Research

The purpose of the present study was to devise an information brochure tailored to the needs and opinions of informal caregivers of persons with DoC. Our study provided initial insights into the usability and acceptability of a brochure prototype, but we have neither implemented it in the care facilities where this study took place nor evaluated it in combination with the verbal communication of physicians. Furthermore, the prototype usability and acceptability were only tested on a small number of purposefully selected informal caregivers; thus, conclusions from the present study cannot be extended to the population of informal caregivers of people with DoC undergoing multimodal assessment. The developed brochure should be further refined during a phase of clinical use and be tested on a representative sample of informal caregivers. Further tests should include objective measures of both health literacy and psychological burden.

Deviations from the theoretical model process [18] guiding the brochure development were also made. These were due to limited time resources and the different objectives of the model process [18] towards the creation of patient decision aids. Decision aids warrant the provision of evidence for different treatment options and therefore are often based on systematic reviews and meta‐analysis. They are constructed to help patients position themselves towards multiple ambiguous treatment options. In contrast, the present brochure was aimed at fulfilling information needs by providing knowledge of multimodal assessment under investigation in the *PerBrain* research project. Moreover, no beta test of the prototype was performed with clinicians to check their perspective on the brochure's acceptability and usability. This was mainly due to the limited number of clinicians who were in principle available in the two research facilities. However, experts with clinical expertise were involved in the steering committee that guided the brochure development. An additional test of the brochure with medical professionals could be subject to future research or be part of its implementation in specific hospitals. Furthermore, the development of a discussion guide specifically addressed to physicians or other healthcare professionals could be considered to take into account the differing extent and complexity of language describing diagnostic measures, background information on DoC, or medical information.

## 5 Conclusion

Applying a participatory development process that integrated perspectives and advice of both the target group of informal caregivers and experts from relevant fields allowed to develop a comprehensive brochure on multimodal assessment of DoC. Other studies testing new (medical) technologies for people with serious illnesses should consider similar measures to give informal caregivers the opportunity to form a comprehensive opinion.

## Author Contributions

**Melissa Hohl:** conceptualization, writing–original draft, investigation, data curation. **Lina Willacker:** conceptualization, writing–original draft. **Theresa Marie Raiser:** conceptualization, writing–original draft. **Martin Justinus Rosenfelder:** conceptualization, writing–review and editing. **Katja Kuehlmeyer:** conceptualization, writing–review and editing. **Marta Bassi:** conceptualization, writing–review and editing. **Angela Comanducci:** conceptualization, writing–review and editing. **Chiara Valota:** conceptualization, investigation, data curation. **Jacobo Diego Sitt:** conceptualization. **Andreas Bender:** conceptualization, writing–review and editing.

## Ethics Statement

This study is part of the EU‐funded, multicentric, international research project *PerBrain: A Multimodal Approach to Personalized Tracking of Evolving State‐Of‐Consciousness in Brain‐Injured Patients*. For the *PerBrain* study, ethical approvals were obtained from all the local ethics committees of the participating study sites (France: ethics committee of the Pitie‐Salpetriere hospital, protocol number M‐Neuro‐DOC, 723 CE SRLF 20‐2; Italy: ethics committee section ‘IRCCS Fondazione Don Carlo Gnocchi’ of ethics committee IRCCS Regione Lombardia, protocol number 32/2021/CE_FdG/FC/SA; Germany: ethics committee of the medical faculty of the Ludwig‐Maximilians‐Universität München, protocol numbers 20‐634 and 20‐635). Written informed consent was obtained from all subjects or their surrogates before entering the *PerBrain* study. For this study, participants were again asked verbally for their consent to participate. Data were collected anonymously.

## Conflicts of Interest

The authors declare no conflicts of interest.

## Supporting information

Supporting information.

Supporting information.

## Data Availability

Within this study, an information brochure for caregivers of persons with DoC was developed. The English prototype brochure version can be found in the Supporting Information for this publication. Translated versions (German, French and Italian) can be made available upon request to the authors. Likewise, raw data from the caregivers' survey and the questionnaire developed and used for the survey are available from the corresponding author upon reasonable request.
