# Supplementary material for: Participatory Development of an International Information Brochure on the Multimodal Assessment of Disorders of Consciousness
Source: Health Expect. 2024 Dec 13;27(6):e70097. doi: 10.1111/hex.70097 (PMC11645296; doi:10.1111/hex.70097)
Supplement: Supplementary file 1 — Supporting information. [file HEX-27-e70097-s001.docx]

Supplementary Material 1

Supplement 1: Results of the scoping review (Table 2)

*Table 2: Results of the narrative review: Study references (column 1); type of interviewed informal caregivers (column 2); Relevant informal caregivers' information needs grouped into the categories Content, Communication style, and Access to information (column 3); implementation of results into the prototype brochure (column 4).*

| **Study** | **Type of informal caregivers** | **Needs of informal caregivers** | **Implementation in the development of the brochure** |
| --- | --- | --- | --- |
|  | **Content** | | |
| ^3–5,56,57^ | Family caregivers | Information about patients’ current condition, diagnosis (results from examinations) and prognosis, as well as potentials and limitations of assessment methods | The developed brochure focuses on clinical diagnosis of DoC, diagnostic tools and their limitations. The prognosis is also part of the brochure. |
| 3 | Family members | Information about expectations of the rehabilitation process, course of care, treatment goals and outcomes, available resources and long-term consequences | This information was not included in the brochure but could be the topic for additional modules. |
| 5 | Family members | Information about the members and working methods of the healthcare team in order to know who the informal caregivers’ contact persons are | This information was not included in the brochure but could be added by the rehabilitation centers. |
| ^5,58^ | Family members | Need to openly address the uncertainty of the diagnosis and prognosis | This topic is addressed in the brochure. |
| ^59^ | Primary family caregivers/ support persons | Difference in type of information requested between the acute phase and long-term rehabilitation | Specific information on different phases of rehabilitation was not included in the brochure but could be the topic for additional modules. |
| 11 | Primary caregivers | Need for clear emphasis on the most important issues. | The brochure offers chapter summaries that are highlighted in color. |
|  | **Communication style** | | |
| 5 | Family members | Repetition of information in different modalities (oral and written) | The brochure offers chapter summaries (written repetition). We recommend that in the informal-caregiver-doctor conversation the content of the brochure will be addressed again (oral repetition). |
| ^4,5,60^ | Family members | Provision of truthful, clear, and consistent information | The brochure offers accurate but general information that should be tailored to the specific conditions of the patients during conversation between informal caregiver and doctor. |
| ^4,5,60^ | Family members | Less technical language | The brochure tries to adopt a clear and straightforward language. Further assessments should evaluate the effectiveness. |
| 3 | Family members | Free communication of information without having to ask for it, as asking is felt as intrusive | The brochure should be offered to every informal caregiver of people with DoC who participate in multimodal assessment. |
|  | **Access to information** | | |
| 14 | Primary caregivers | Accessibility of health information through in-person communication or via internet | The brochure should be distributed by medical personnel as well as be freely accessible on the internet |
| 14 | Primary caregivers | Information supply through support groups | This is an opportunity that could be considered in the future. Yet, it could also raise false expectations since multimodal assessment is only offered in few rehabilitation facilities and its outcomes are currently under investigation. |

Supplement 2: Informal caregivers’ opinions of the brochure chapters (Table 3)

*Table 3: Summary of caregivers’ qualitative evaluation of the brochure prototype. Feedback was provided by informal caregivers during the beta testing.*

| **Chapter** | **Caregivers’ evaluations** |
| --- | --- |
| Chapter 1: Definition and meaning of DoC | Participants rated this chapter as helpful in understanding what DoC are and expressed that it was easy to understand. Yet, some medical terms and paragraphs were particularly difficult to understand. Ten participants stated that the abbreviations used in this chapter were not easy to grasp and should be removed. Overall, participants reported that they understood the main messages the chapter intended to convey. |
| Chapter 2 |  |
| Chapter 2.1: What are opportunities and challenges in neurodiagnostics? | Answers to open-ended questions showed that verifying whether physicians were guided by the diagnostic criteria portrayed in the brochure was difficult for two participants in Germany (2/6). One caregiver rated the list of criteria that doctors should consider when making a diagnosis as helpful. Ten participants indicated that a figure on diagnostic measures should highlight which ones are available everywhere and which only in some centers. Several participants further expressed that the introduction should clearly state that the brochure is not a guideline nor a general recommendation. |
| Chapter 2.2: How do doctors make the diagnosis? | An illustration showing which diagnostic measure is used for which question (e.g., hidden awareness) was rated as not very helpful and difficult to understand (10/12) since too many abbreviations were included and some of the listed methods were not available everywhere. One participant stated: *"The outline is too difficult for me, thinking that a family member is reading it and not a doctor. I don't know how comprehensible it is and how memorable it is. I also don't understand why some measures turn out to be present in only some centers. This worries me as a family member, and I don't really understand why a measure is present or not in a center"*. On the other hand, one participant thought the illustration was well designed to work with the information and to get an overview of which medical centers offered which methods. |
| Chapter 2.3: Which neurodiagnostic methods are available? | Participants mentioned that some measures were presented too technically and in too much detail. Illustrations of the measures were considered as helpful. The chapter summary was difficult to understand because it contained too many abbreviations. The conditions required for the specific measures should be clearly listed in the summary. The use of the term "probably yes" in the summary was judged as not very useful, even if it was in line with current scientific knowledge. |
| Chapter 3: Capabilities of people with DoC according to their diagnosis | The different DoC classification categories (coma, UWS, MCS), the explanations about the behavior people with DoC can show, and the questions at the end of the chapter were judged as particularly helpful. One participant stated*: "The sentence ‘A patient has exited MCS when he or she is able to communicate in an intentional and reproducible way, verbally or nonverbally, or when he or she is able to use objects functionally.’ - It was very confusing to me, as it is difficult for me to understand, for example, what is meant by nonverbal communication in a patient who does not move, or in any case, it is difficult for me to understand how exit from MCS can happen"*. Similarly, the formulation of the sentence "*By definition, the MCS patient cannot communicate consistently either verbally or nonverbally"* was perceived as incomprehensible and confusing. Nonetheless, the main message of the chapter was rated as understandable and the usefulness, comprehensibility and information content of this chapter were rated positively. |
| Chapter 4: Implications of results from multimodal assessment | This chapter was divided into three sections (4.1: “What is the relevance of diagnostic test results for prognosis?”, 4.2: “When does a DoC become chronic?”, 4.3: “What is the difference between regained consciousness and functional/motoric independence?”), but they were evaluated together. The sentence *"A study from Germany has shown that up to 1/3 of patients emerge from an MCS"* in section 4.3 was not understandable or potentially frustrating. Caregivers wished that the difference between functional and motor independence be emphasized more. One of them said: *"It is really important to explain that it is not like in movies the patient just wakes up and can walk and have their past life back*". Yet one caregiver expressed skepticism about the brochure’s supposed use. To the question *“Does the brochure help with the conversation?”* the caregiver answered*: "Rather not, communication depends strongly on the people or the particular ward if they feel like explaining a lot or not".* |
| Chapters 5 and 6 (Outlook) | Here, information boxes, especially containing webpages, were desired. |

Supplement 3: Caregivers’ quantitative ratings of the tested brochure prototype (Table 4)

*Table 4: Summary scores for the brochure testing. The following answering format was applied: 1= Strongly disagree, 2= Do not agree, 3= Somewhat disagree, 4= Somewhat agree, 5= Agree, 6= Strongly agree. IQR = interquartile range, displayed as third minus first quartile (Q3-Q1). The first and third quartile are denoted in brackets. The total number of participating caregivers was 12. Questions that were only answered by 11 caregivers are marked by *.*

| **Question** | **Median** | **IQR [Q1 Q3]** |
| --- | --- | --- |
| ***Chapter 1*** |  |  |
| The explanations in chapter 1: “My loved one has a disorder of consciousness – what does that mean?” are helpful to understand the condition. | 5 | 0 [5 5] |
| The information in chapter 1: “My loved one has a disorder of consciousness – what does that mean?” is easy to understand. | 5 | 1 [4 5] |
| The amount of information in chapter 1: “My loved one has a disorder of consciousness – what does that mean?” is sufficient to clarify your questions. | 5 | 1 [4 5] |
| ***Chapter 2*** |  |  |
| The explanations in section 2.1: “What are the opportunities and challenges in neurodiagnostic” are helpful to better define one's own expectations for diagnostics. | 4.5 | 1 [4 5] |
| The information in section 2.1: “What are the opportunities and challenges in neurodiagnostic” is easy to understand. | 4 | 2.5 [3.25 5.75] |
| The amount of information in section 2.1: “What are the opportunities and challenges in neurodiagnostic” is sufficient to clarify your questions. | 5 | 1 [4 5] |
| The explanations in section 2.2: “How can the doctors proceed when making the diagnosis” are helpful to understand the diagnosis process. | 5 | 2.75 [3.25 6] |
| The information in section 2.2: “How can the doctors proceed when making the diagnosis” is easy to understand. | 4 | 1.75 [3.25 5] |
| The information in section 2.2: “How can the doctors proceed when making the diagnosis” is sufficient to clarify your questions. | 4 | 1 [4 5] |
| The explanations in section 2.3: “Which neurodiagnostic methods are available?” are helpful to answer this question. | 5* | 2 [3 5] |
| The information in section 2.3: “Which neurodiagnostic methods are available?” is easy to understand. | 4 | 2 [3 5] |
| The information in section 2.3: “Which neurodiagnostic methods are available?” is sufficient to clarify your questions. | 5* | 2 [4 6] |
| ***Chapter 3*** |  |  |
| The explanations in chapter 3: “What can I expect from my loved one?” are helpful to get an overview of the possible reactions. | 5 | 1.5 [4.25 5.75] |
| The information in chapter 3: “What can I expect from my loved one?” is easy to understand. | 5 | 1 [4 5] |
| The information in chapter 3: “What can I expect from my loved one?” is sufficient to clarify your answers. | 5 | 1 [4 5] |
| ***Chapter 4*** |  |  |
| The explanations in section 4.1: “What is the relevance of diagnostic test results for prognosis?” are helpful to understand this connection. | 4.5 | 1 [4 5] |
| The information in section 4.1: “What is the relevance of diagnostic test results for prognosis?” is easy to understand. | 4* | 1 [4 5] |
| The information in section 4.1: “What is the relevance of diagnostic test results for prognosis?” is sufficient to clarify your questions. | 5 | 1 [4 5] |
| The explanations in section 4.2:” When does a DoC become chronic?” are helpful to answer this question. | 4 | 1 [4 5] |
| The information in section 4.2:” When does a DoC become chronic?” is easy to understand. | 5 | 1 [4 5] |
| The information in section 4.2:” When does a DoC become chronic?” is sufficient to clarify your questions. | 4 | 1 [4 5] |
| The explanations in section 4.3:” What is the difference between regained consciousness and functional/motoric independence?” are helpful to answer this question. | 5 | 1.8 [4 5.8] |
| The information in section 4.3:” What is the difference between regained consciousness and functional/motoric independence?” is easy to understand. | 5 | 2 [4 6] |
| The information in section 4.3:” What is the difference between regained consciousness and functional/motoric independence?” is sufficient to clarify your questions. | 5 | 1 [4 5] |
| ***Chapter 5*** |  |  |
| The explanations in chapter 5:” Other topics that could be of interest” are helpful to gain an overview. | 5.5 | 1 [5 6] |
| The information in chapter 5: Other topics that could be of interest” is sufficient to clarify my questions. | 5.5 | 1 [5 6] |
| ***Chapter 6*** |  |  |
| The explanations in chapter 6: “Recommendations for more information” are helpful to assist me in my search. | 5 | 1 [4.3 5.75] |
| The information in chapter 6: Recommendations for more information” is sufficient to clarify my questions. | 4.5 | 2.8 [2.3 5] |
| ***Layout & Design*** |  |  |
| The font (Arial 13) is large enough to read | 6 | 1 [5 6] |
| The brochure has the right format size (DIN A4) | 6 | 1 [5 6] |
| The order of contents make sense | 5 | 1.5 [4.5 5.8] |
| There is too much text on one page for your liking | 3.5 | 3.8 [1.3 5] |
| The illustrations help to understand the text better | 5.5 | 1.8 [4.3 6] |
| The chapter summaries are useful | 5 | 1 [5 6] |
| The space for notes is useful | 5 | 3.8 [2.3 6] |
