# Supplementary material for: Participatory Development of an International Information Brochure on the Multimodal Assessment of Disorders of Consciousness
Source: Health Expect. 2024 Dec 13;27(6):e70097. doi: 10.1111/hex.70097 (PMC11645296; doi:10.1111/hex.70097)
Supplement: Supplementary file 2 — Supporting information. [file HEX-27-e70097-s002.docx]

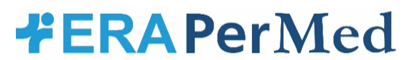
 Logo care facility

**Coma or consciousness?**

How to test consciousness in patients with severe brain injury

A brochure for family members and friends of patients with a disorder of consciousness

This information brochure is a result of the EU-funded, international research project “EraPerMed – PerBrain”. It was developed by researchers with regular consultation of an advisory board, as listed below.

**Authors**

| - Prof. Dr. Andreas Bender ^1,2^ - Dr. Lina Willacker ^1^ - Melissa Hohl ^1^ - Dr. Theresa M. Raiser ^1^ |  |
| --- | --- |

Institutions:
^1^ Department of Neurology, LMU University Hospital, LMU Munich, Munich, Germany
^2^ Therapiezentrum Burgau, Hospital for Neurological Rehabilitation, Burgau, Germany

**Advisory board**

- Dr. Angela Comanducci (IRCCS Fondazione Don Carlo Gnocchi ONLUS, Milan, Italy)
- Prof. Dr. Anke Steckelberg (Department of health and nursing sciences, Martin Luther University Halle-Wittenberg, Germany)
- Dr. Chiara Valota (University of Milan, Italy)
- Dr. Chiara-Camilla Derchi (IRCCS Fondazione Don Carlo Gnocchi ONLUS, Milan, Italy)
- Dr. Katja Kuehlmeyer (Institute of Ethics, History and Theory of Medicine, LMU Munich, Germany)
- Marit Hole (Therapiezentrum Burgau, Hospital for Neurological Rehabilitation, Germany)
- Prof. Marta Bassi (University of Milan, Italy)
- Martin Rosenfelder (Therapiezentrum Burgau, Hospital for Neurological Rehabilitation, Germany)
- Paola Emilia Cicerone (Freelance science writer, Italy)
- Dr. Pietro Davide Trimarchi (Neuropsychologist, Italy)
- Prof. Dr. Ralf J. Jox (Lausanne University Hospital, Switzerland)
- Reinhard Schaler (An Saol Neurorehabilitation Day Centre, Dublin, Ireland)
- Robert Förg (Therapiezentrum Burgau, Hospital for Neurological Rehabilitation, Germany)

Dear Reader,

caring for someone with a disorder of consciousness due to a severe brain injury is a difficult task. The care can involve persons like you, family members, friends, and also professional caregivers. To better understand the conditions patients with a disorder of consciousness are in, this brochure provides you with selected information about the medical assessment of consciousness. It has been created within the research project “EraPerMed – PerBrain” which investigates the effectiveness of diagnostic measures and their implications for medical care for patients with a disorder of consciousness. Its content has been discussed in a transdisciplinary team which included physicians, clinical psychologists, experts in medical ethics, neuropsychologists, health communication professionals, and engaged caregivers.

**The aim of this brochure** is to provide information about disorders of consciousness and how to measure consciousness with the help of partly new technological measures to support diagnostics and prognostics. It is not a guideline or recommendation for diagnosis. Remember that more measurements are not necessarily better. Its purpose is to facilitate and accompany the communication process between you and your health care team. This brochure cannot substitute communication with health professionals.

**Usage tips**: You can use it to prepare for doctor appointments, doctors can refer to it during these conversations, and you can use it after the appointments to reflect on the conversation. When you find this symbol
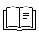
, you can get more information about the related topic in the glossary at the end of the brochure (p.32).

We hope, the brochure can help you gain a comprehensive understanding of the background of the diagnosis of disorders of consciousness.

**Table of Contents**

[1 My loved one has a disorder of consciousness - what does that mean? 1](#_Toc130798008)

[1.1 What is the cause and what are the effects? 1](#_Toc130798009)

[1.1.1 What structural consequences in the brain can arise? 1](#_Toc130798010)

[1.1.2 What functional consequences can arise? 2](#_Toc130798011)

[1.2 Diagnostic categories of DoC 3](#_Toc130798012)

[2 What are the recent diagnostic tools for disorders of consciousness? 7](#_Toc130798013)

[2.1 How do doctors make the diagnosis? 7](#_Toc130798014)

[2.2 Which neurodiagnostic methods are available? 9](#_Toc130798015)

[2.2.1 Structured observational methods – use of scales 9](#_Toc130798016)

[2.2.2 Technical methods – use of measuring instruments 10](#_Toc130798017)

[Functional magnetic resonance imaging (fMRI) 10](#_Toc130798018)

[Electroencephalography (EEG) 12](#_Toc130798019)

[High Density- Electroencephalography 13](#_Toc130798021)

[Transcranial Magnetic Stimulation - Electroencephalography 14](#_Toc130798022)

[Positron-Emission-Tomography (PET) 16](#_Toc130798023)

[Nasal airflow measurement via “Moustache” 17](#_Toc130798024)

[2.3 What are the opportunities and challenges in neurodiagnostics? 19](#_Toc130798025)

[3 Different behaviours in all states of disorder of consciousness 24](#_Toc130798026)

[4 Disorders of consciousness and the assessment results – what does it mean for my loved one in the future? 27](#_Toc130798027)

[4.1 What is the relevance of diagnostic test results for prognosis? 27](#_Toc130798028)

[4.2 When does a disorder of consciousness become chronic? 27](#_Toc130798029)

[4.3 What is the difference between regained consciousness and functional/motor independence? 28](#_Toc130798030)

[5 Other topics that could be of interest 31](#_Toc130798031)

[6 Recommendations for more information 32](#_Toc130798032)

# My loved one has a disorder of consciousness - what does that mean?

Consciousness is the state of being awake and aware of one's surroundings. We talk about disorders of consciousness when someone has difficulty maintaining wakefulness and/or is suffering from impaired awareness of themselves and their surroundings due to brain injury.

## What is the cause and what are the effects?

Disorders of consciousness can occur when the corresponding networks in the brain responsible for consciousness are injured. This can be due to either:

- A direct traumatic injury to tissue in the skull, especially in the brain, most often caused by falls, traffic accidents, or violent assaults, or
- A non-traumatic brain injury, for example stroke, cardiac arrest, infection, or chronic disease

### What structural consequences in the brain can arise?

After brain injury, the damage to the brain tissue may be direct and/or delayed due to swelling and bleeding caused by the injury. The tissue inside the skull can be divided into three parts: brain tissue
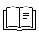
, blood, and liquor
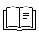
. If one of these three parts enlarges due to the injury, it is to the detriment of the other two. This structural change can be visualised with the help of brain scans. The resulting damage to the brain tissue is specific for each patient.

### What functional consequences can arise?

The functional consequences are highly subjective and have different levels of severity in patients. As a rule of thumb, functional impairments can affect 3 main areas:

| **Motor functions**   - Reflexes such as blinking or yawning - Automatic movements such as swallowing - Voluntary limb movements | **Sensory functions**   - Hearing sounds - Feeling touch - Feeling pain - Sight - Taste | **Complex cognitive functions** (please note that only few functions are reported for explanatory purposes)   - Perception - Attention - Memory - Speech - Visuospatial ability - Executive functions |
| --- | --- | --- |

## Diagnostic categories of DoC

Disorders of consciousness can be divided into three main categories:

Key Point: Brain death, Locked-in-Syndrome and emerged MCS probably do not concern your relative

- **Coma**: An acute condition which usually lasts only a few days or weeks. It can be interpreted as a state of deep unconsciousness from which the patient cannot be awakened. The patient shows no alertness or intentional responsiveness to any kind of stimulation with the exception of reflex activities which may be variably spared. The eyes are closed, and the normal sleep-wake cycle is disrupted.
- **Unresponsive Wakefulness Syndrome (UWS)**: the patient reopens his or her eyes (i.e., regains a state of wakefulness) but a state of unresponsiveness remains. No clinical signs of consciousness could be detected (i.e., not responding to stimuli at the verbal or motor level). Even if the eyes can be opened, the patient usually shows only reflexive activities (for example a cough reflex when swallowing). In this condition, a patient is therefore considered unaware of himself and the surrounding environment. A popular synonym for Unresponsive Wakefulness Syndrome is Vegetative State (VS).

controlled movement

- **Minimally Conscious State (MCS)**: a state characterised by an improvement of responsiveness, in which the patient shows more than purely reflexive behaviour as observed in Unresponsive Wakefulness Syndrome. Once a patient has transited into a condition of minimally conscious state, it is difficult to come up with a final prognosis as to their potential. Indeed, progresses are possible even after a long period of time from the brain injury. When needed, it is important to continue with adequate neurorehabilitation activities, especially in the acute-subacute phase also to prevent secondary injuries. Depending on the degree of behavioural response, patients are further subdivided into:

Eye opening

- - **MCS- ("minimally conscious state minus”)**: The patient shows only some signs of conscious responses, such as pain localisation, e.g., when the arm is pressed too hard, or when they can visually track people or objects in the room. They might react, but they do not respond in a reproducible way, to motor commands. No behaviours indicative of language understanding, and processing can be observed in a minimally conscious state- patient.
  - **MCS+ ("minimally conscious state plus”)**: The patient can follow simple commands, sometimes they can also produce understandable words, and/or demonstrate intentional communication.

A synonym for minimally conscious state could be minimally responsive state.

When diagnosing disorders of consciousness, the two following conditions must be excluded:

- **Brain death**: Irreversible loss of all brain function. For this, different criteria must be met, which are defined by law. In contrast to disorders of consciousness, the patient has no brainstem reflexes and is not able to breathe spontaneously.
- **Locked-in-Syndrome:** this syndrome involves an almost complete paralysis of the majority of muscles of the body (also face and trunk) which could be voluntarily controlled. However, in this case consciousness and mental functions are not affected. The patient is not able to produce facial expression, move their body, speak, or verbally communicate, but they can commonly move their eyes up and down and blink. Therefore, these residual eye movements can be employed to communicate.

In addition to the diagnostic categories, there is another important condition to know, the **cognitive motor dissociation (or covert consciousness).** This concerns the patient who does not show any reaction at the behavioural examination. However, during investigational examinations, e.g., with the Functional magnetic resonance imaging (fMRI) or Electroencephalography (EEG), they show a command response that is not visible on behavioural examination.


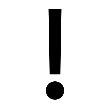


**Summary of Chapter 1 – What does a disorder of consciousness mean?**

- Following a brain injury, every patient has functional and structural consequences which are specific and different at single-person level.

Appearance of eye opening

-
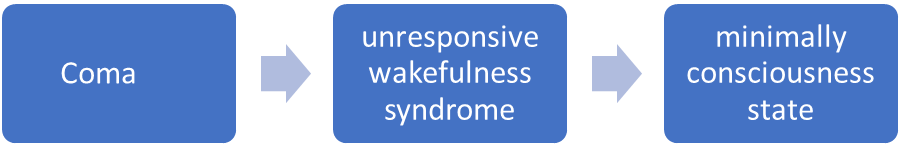
There are 3 main types of disorders of consciousness: Coma, unresponsive wakefulness syndrome, and minimally consciousness state

Appearance of controlled or voluntary movement

- Brain death and Locked-in-Syndrome do not fall within a diagnosis of disorder of consciousness and are different conditions.


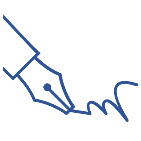
**My notes for chapter 1 – What would I like to ask?**

If you have found anything unclear and have questions while reading the chapter, you can note them down below and ask your doctor during a conversation.

# What are the recent diagnostic tools for disorders of consciousness?

Specific diagnostic tools can help us to better understand disorders of consciousness and a patient’s current condition. Moreover, they can be used to make better predictions about the future development of the patient.

## How do doctors make the diagnosis?

The usual way of proceeding in the neurodiagnostic assessment of disorders of consciousness is a two-pronged approach. A clinical assessment (Coma Recovery Scale-Revised) is performed at the bedside as standard practice. The results of this assessment can be complemented by innovative neuroimaging (such as magnetic resonance imaging) and neurophysiological techniques (such as electroencephalography), where the clinician will obtain more objective data of brain function. Depending on their purpose, complexity, and availability, neurodiagnostic tools can be divided into 4 categories [3]. In the following figure, you can see which method can be used for which question that doctors ask themselves (e.g., what is the extent of brain damage?). Each method, with its advantages and disadvantages, is described in detail in paragraph 2.2.3. Since these are partly new and innovative methods, not every hospital has the necessary technical equipment for each category. If you want, you can mark the assessments that can be performed in your medical centre. But keep in mind that more measurements are not necessarily better.

EEG= Electroencephalography
MRI= Magnetic resonance imaging
fMRI= functional magnetic resonance imaging
PET= Positron-Emission-Tomography
TMS-EEG= Transcranial Magnetic Stimulation – Electroencephalography

## Which neurodiagnostic methods are available?

This brochure focuses on the instrumental methods: functional magnetic resonance imaging, electroencephalography, Transcranial Magnetic Stimulation - electroencephalography, Positron-Emission-Tomography, nasal airflow measurement, and clinical behavioral assessment (CRS-R). However, it is possible that in other hospitals other methods, such as functional Near-Infrared Spectroscopy (fNIRS) or blood markers, are employed. In this case, do not hesitate to ask your doctor for further information.

### Structured observational methods – use of scales

#### **Coma Recovery Scale-Revised (CRS-R)**

Currently the Coma Recovery Scale-Revised (CRS-R) is considered the reference for the diagnosis of disorders of consciousness and for quantifying the degree of behavioural responsiveness and is therefore available in all clinics. This scale measures the responsiveness of the patient with impaired consciousness in relation to five different domains (auditory, visual, motor, oromotor, and communication) as well as in relation to their general level of consciousness. The assessment can take place at the bedside. The physician / neuropsychologist uses a range of defined tasks and commands to assess the patient’s residual behavioural capacities in the different explored domains: for example, how the patient reacts to sounds, if they can recognise and use everyday objects or communicate with others using either voice or certain body movements (e.g., tongue, finger, head).

Benefits of this method: The Coma Recovery Scale-Revised can be used to establish a diagnosis, to monitor the recovery, to predict outcomes, and to evaluate the effectiveness of treatments in relation to consciousness.

Limitations of this method:

- The administration of the scale is time consuming (15-60 minutes), and it requires experienced and trained personnel.

For these reasons, in special settings such as the intensive care units or whenever the time for performing evaluations is limited, other assessment scales are used for a regular monitoring.

### Technical methods – use of measuring instruments

### Functional magnetic resonance imaging (fMRI)

Functional magnetic resonance imaging (fMRI) can be used to visualise the level of activity in the brain at rest and while performing a task. It can also help to distinguish active areas of the human brain from inactive ones. Depending on what the patient is doing during the examination, the active areas can then be assigned to different functions (such as speech, memory, movements). Doctors refer to these as "functional areas."

Functional magnetic resonance imaging scans create slice images of the brain without using X-rays
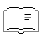
. Instead, these images are produced using a magnetic field and radio waves
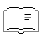
. Nerve cells in active brain areas need nutrients - oxygen and glucose. To supply this demand, a complex reaction between the vessels and the nerve cells leads to an increase in blood flow to the activated brain area. The oxygenated blood can bring about changes in the local magnetic field, which can be detected using functional magnetic resonance imaging. Through complex post-processing of the image data acquired, these changes can be displayed and identified as activity of the brain area.

During functional magnetic resonance imaging measurements, the patient lies on their back on a cushioned table (Fig. 1), which moves into the magnetic resonance imaging scanner (tube). To obtain an image at a resting state, the patient must lie still. For an active exposure, tasks (e.g., “move right hand”) are set, or the patient experiences a stimulus (e.g., visual, or electrical). This takes half an hour on average. Functional magnetic resonance imaging measurements are not invasive and are painless.


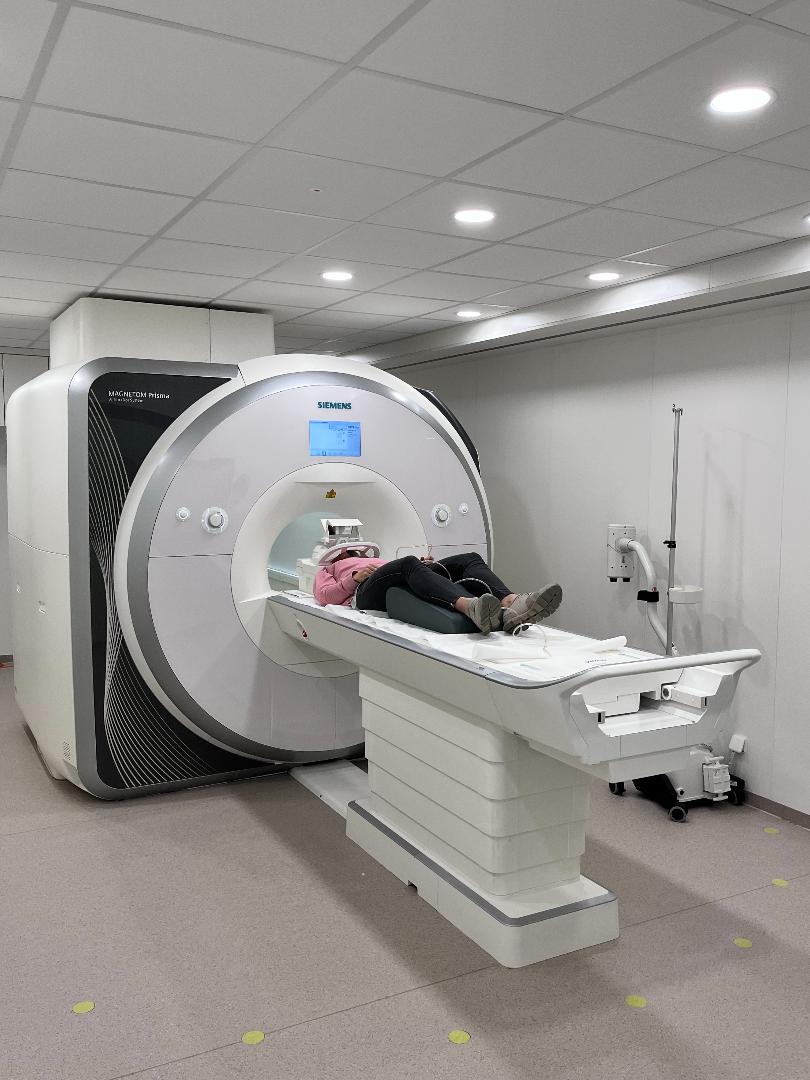


Figure 1: The patient lies on a cushioned table, which moves into the magnetic resonance imaging scanner (tube)

Benefits of this method: the functional magnetic resonance imaging can detect brain activation patterns, even for patients who are not able to respond to commands during behavioural assessment or who do not show any overt behavioural reactions.

Limitations of this method:

- Brain activation following commands detected by means of functional magnetic resonance imaging does not necessarily relate to consciousness because the brain can also respond to a stimulation while the person is not aware of it. A brain activation pattern that proves consciousness has not yet been defined
- On the other hand, absence of activity shown by functional magnetic resonance imaging in certain brain areas or during specific tasks is no proof of absence of consciousness
- Functional magnetic resonance imaging cannot be used in patients who are clinically unstable, unable to be transported to the scanner, have implanted ferromagnetic material, or make frequent head and body movements
- The need to bring patients to the scanner makes repeated assessments difficult

### Electroencephalography (EEG)

For electroencephalography (EEG) measurements, small electrodes are placed on the head, usually with the aid of a cap (Fig. 2), to record the electrical signals that are generated by the activity of the brain cells under the skull. Electroencephalography provides information about the activity of the brain. As with functional magnetic resonance imaging, electroencephalography can be measured at rest or during an activity, for example when looking at pictures or during sleep. There are different types of electroencephalography, which are presented below. They have in common that the recording can take place at the bedside, thus making it possible to perform several tests at different states of arousal (e.g., resting, or active state). Electroencephalography measurements are painless, non-invasive and can be carried out in almost all patients, even in those with ferromagnetic implants such as a pacemaker.

**Standard Electroencephalography**

Standard Electroencephalography complements behavioural and imaging assessment of disorders of consciousness. It is usually performed while the patient rests and is not involved in any specific activity. Standard electroencephalography can help to:

- Determine the extent of the acquired brain damage
- Formulate an initial prognosis if the electrical activity of the brain is significantly suppressed
- Detect a global preservation of electroencephalography background activity suggesting a locked-in syndrome


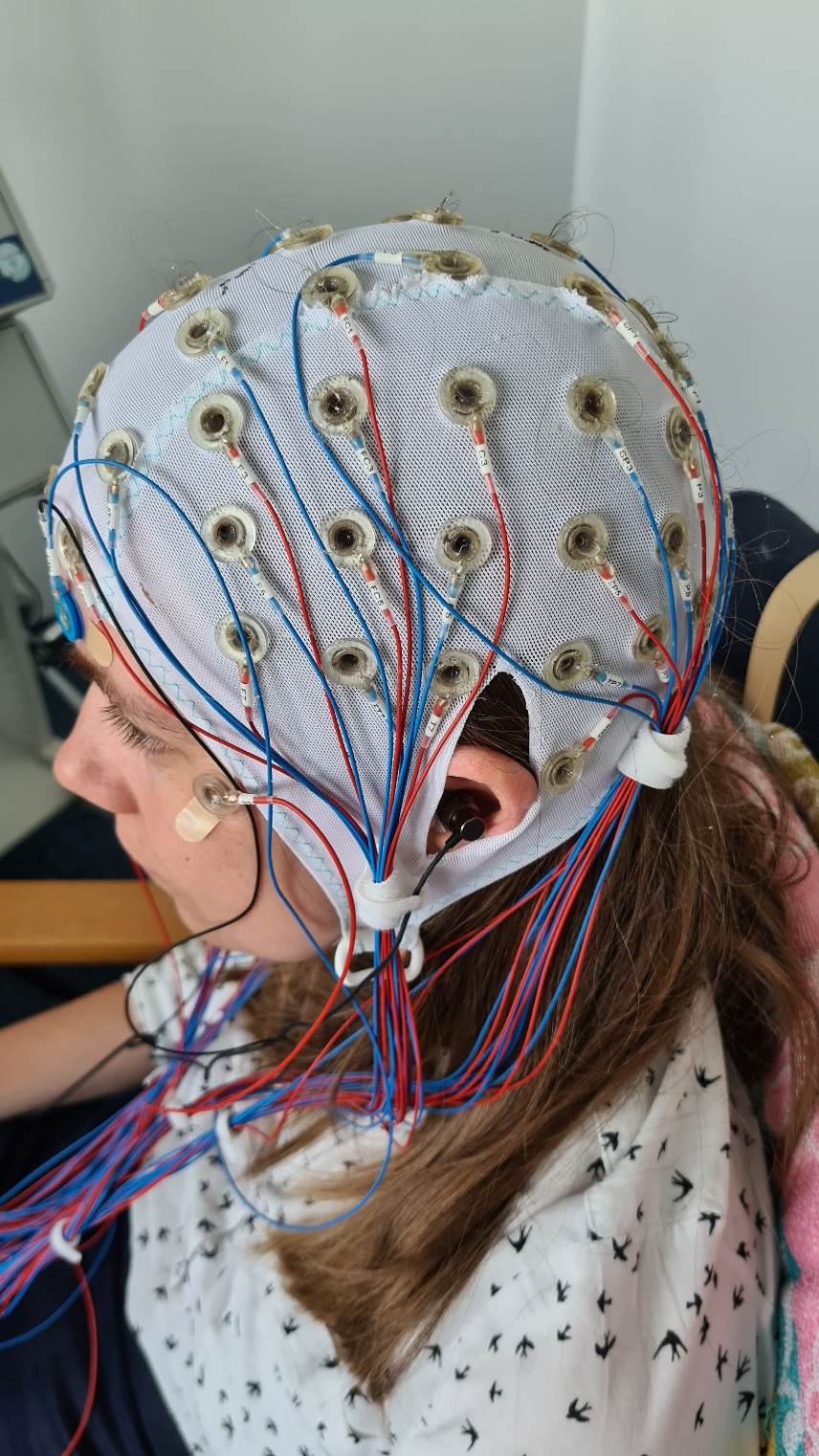

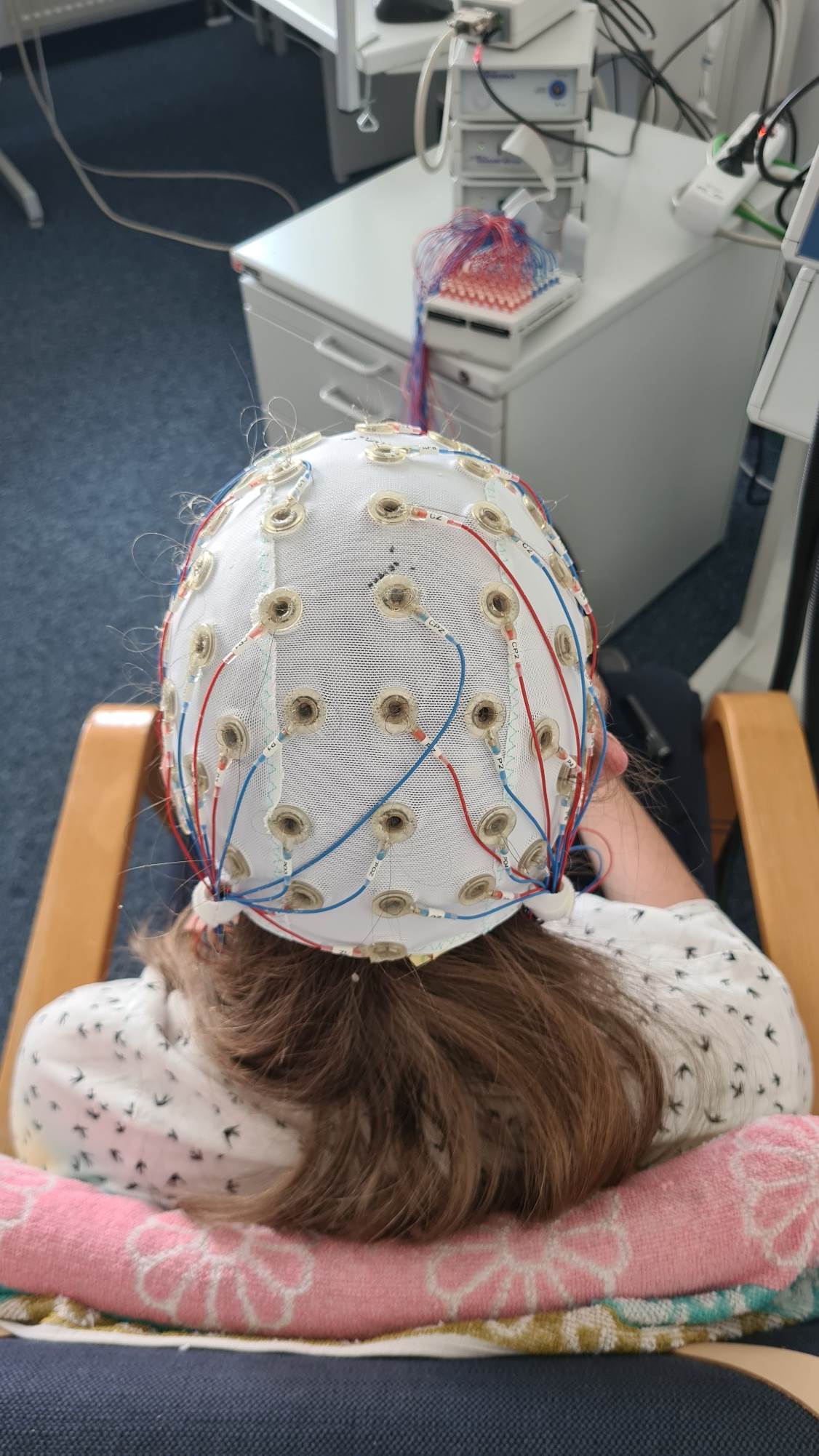


Figure 2: Electrode cap for electroencephalography measurement

Figure 3: Electroencephalography measurement with recording equipment

### High Density- Electroencephalography

In terms of structure and principle, the high-density electroencephalography (HD-EEG) corresponds to the standard electroencephalography. The only difference is that for high-density electroencephalography more electrodes are used for the measurement (Fig. 3). As with standard electroencephalography, high-density electroencephalography can be used at rest. Alternatively, it is often applied in combination with an activity or while the patient is presented with different stimuli (e.g., sounds or pictures). Such active electroencephalography paradigms
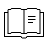
 can be used to measure brain responses during an activity or in reaction to stimuli. In that way, residual cognitive abilities, which are not observable to the eye, can be detected with the help of electroencephalography.

Benefits of this Method:

- Better differentiation between unresponsive wakefulness syndrome and minimally consciousness state
- Active electroencephalography (use of activities or stimuli) can identify cognitive-motor dissociations: patients who can imagine performing tasks even though they appear completely unresponsive during the bedside behavioural assessment

Limitations of this method: Because of the lack of a general agreement for the classification of disorders of consciousness (coma, unresponsive wakefulness syndrome, minimally consciousness state, c.f. paragraph 1.2), an accurate assessment of consciousness based on (high-density) electroencephalography is not possible yet.

### Transcranial Magnetic Stimulation - Electroencephalography

While methods like functional magnetic resonance imaging or electroencephalography measure brain activity, transcranial magnetic stimulation (TMS) can directly influence the activity of the brain. Very short electrical pulses are sent through the skull via a magnetic coil which is held on top of the patient’s head. In so doing, the natural activity of the stimulated brain region is modified for a short time. For example, if the motor cortex, an area which is mainly responsible for movement, is stimulated with TMS, a muscle twitch is triggered. Transcranial magnetic stimulation is not invasive and is largely painless for the patient. To record the brain's response to the stimulation, Transcranial-magnetic-stimulation can be combined with concurrent electroencephalography measurements (Fig. 4).


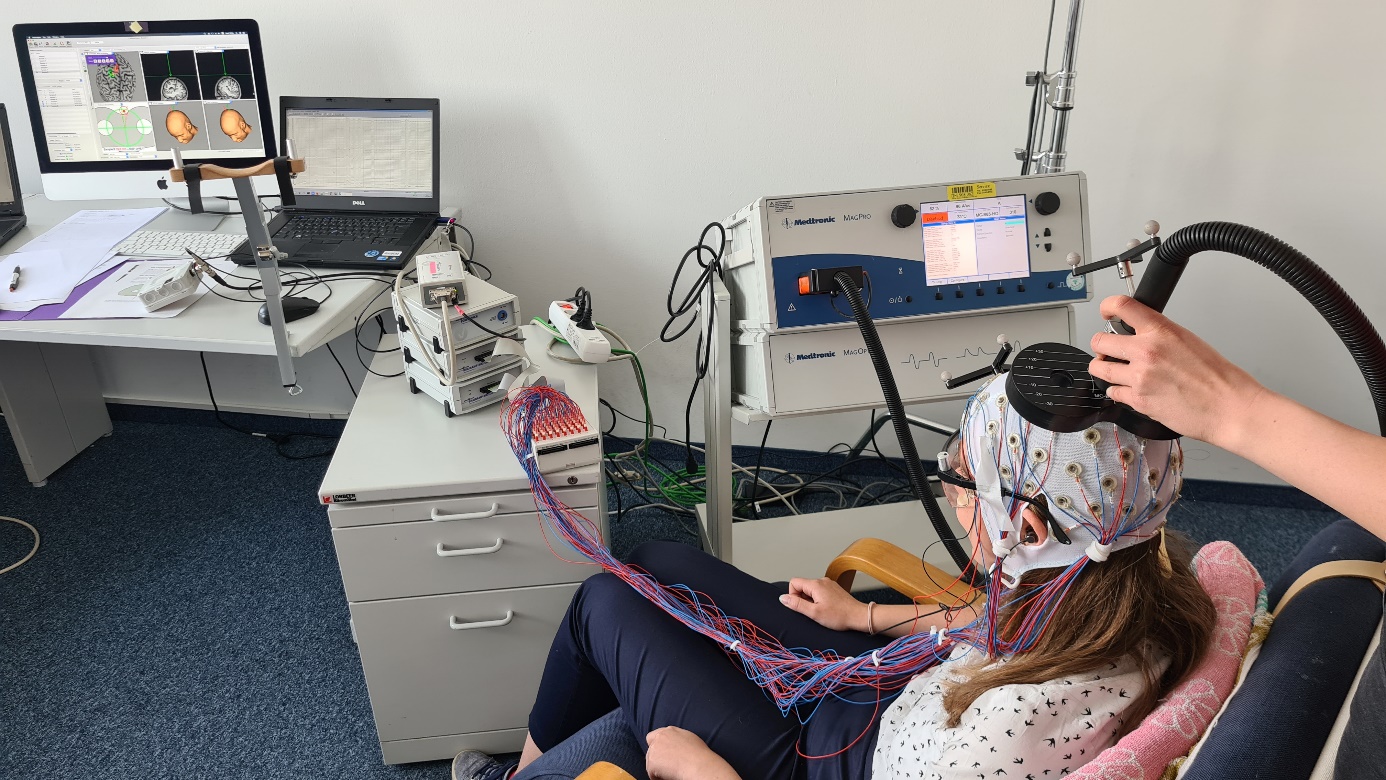


Figure 4: Transcranial Magnetic Stimulation - Electroencephalography examination

Benefits of this Method:

- Transcranial Magnetic Stimulation – Electroencephalography recordings can be performed at the bedside, without direct patient participation or speech understanding
- Transcranial Magnetic Stimulation - Electroencephalography allows for the direct stimulation of the specific brain areas of interest and influences their activity
- Better differentiation between unresponsive wakefulness syndrome and minimally consciousness state

Limitations of this method:

- Transcranial Magnetic Stimulation cannot be applied in patients with ferromagnetic implants near the head
- Transcranial Magnetic Stimulation cannot apply if a bone flap of the skull is removed
- Transcranial Magnetic Stimulation – Electroencephalography measurements are time-consuming and require well-trained personnel

### Positron-Emission-Tomography (PET)

Positron-Emission-Tomography measurements can show how active certain areas of the brain are by visualising their metabolic activity. The measurements involve taking several images of the brain that show the region under investigation layer by layer, as if in thin slices. A computer then generates a three-dimensional image from these slices.
To make the metabolic processes visible, a very weak radioactive substance (also called a "tracer") is used, which is usually injected into the arm vein. The tracer connects to glucose
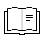
. The connection of tracer and glucose is absorbed from the blood into the cells like normal sugar and used to produce energy. The radiation produced when the tracer decays can be detected by a scanner. This makes cells or tissues that consume a lot of energy and have a high metabolic rate particularly visible. Tissues with a fast metabolic rate and high sugar consumption appear as particularly dark spots on black-and-white Positron-Emission-Tomography images, or as particularly bright spots on colour images. Thus, Positron-Emission-Tomography scans can be used to show which areas of the brain are active. In a minimally consciousness state, higher metabolic activity is to be expected in the areas of the brain that are important for consciousness.

Benefits of this method:

- Positron-Emission-Tomography has the potential to differentiate between unresponsive wakefulness syndrome and minimally consciousness state
- Early detection of patients with relative preservation of brain metabolism Lower the risk of premature withdrawal of life-sustaining therapy.

Limitations of this method:

- Confounding factors (e.g., diabetes, epilepsy) should be taken into account, as they may bias results; high technical standards are necessary to control this possibility
- During the injection of the radioactive tracer the patient must be sufficiently aroused
- Exposure to radiation, albeit limited, is necessary.

### Nasal airflow measurement via “Moustache”

Sense of smell relies on deep brain structures that are involved in basic mechanisms of arousal. It is tested by accurately measuring odorant-dependent (good/bad/non-smell) breath volume. In particular, the nasal airflow of patients is measured using a nasal cannula (“Moustache”) linked directly to a spirometer
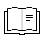
 (Fig. 5). If the patient changes their breathing according to the smells, it will show in the breathing frequency. So, it becomes visible how the patient perceives this smell and how they are conscious or not. For that measurement it is not necessary for the patient to speak or move.


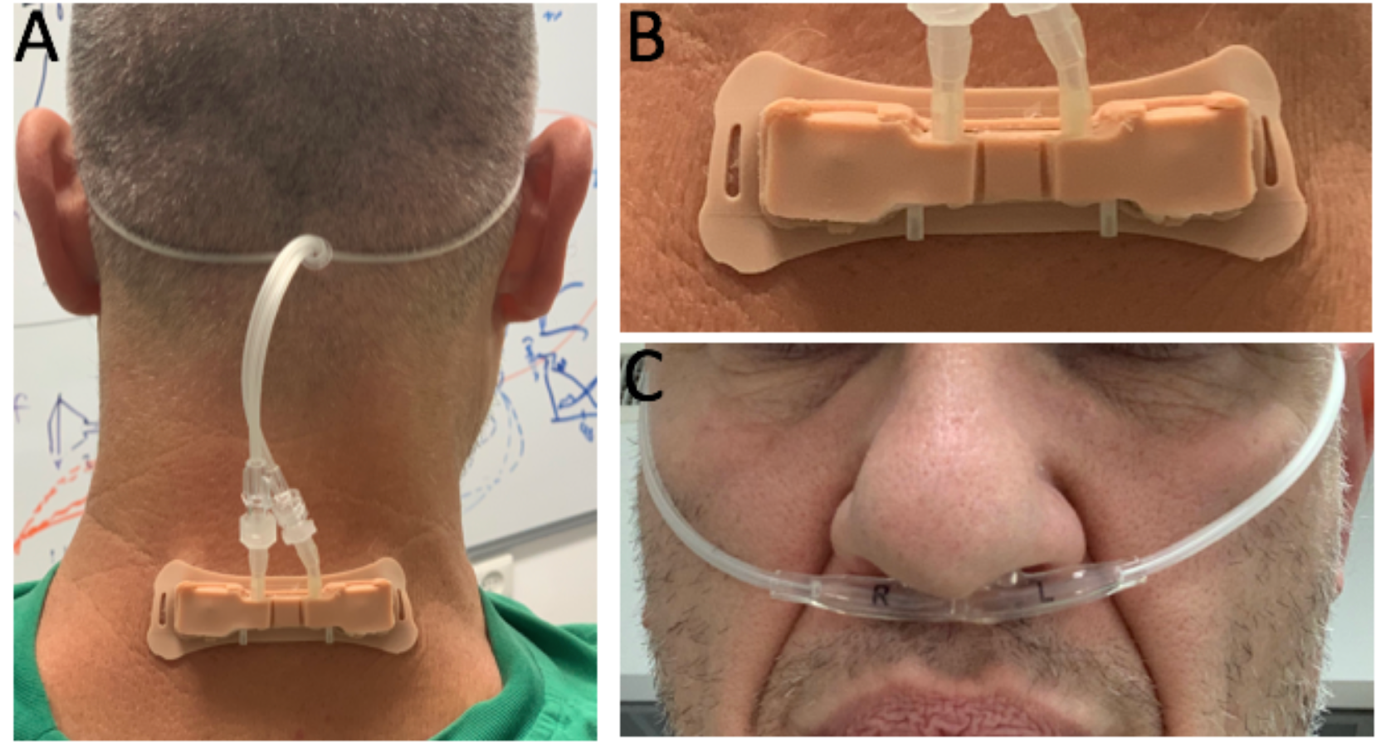


Figure 5 A-C: The nasal cannula is attached to the patient for the measurement of odorant-dependent breath volume.

Benefits of this Method:

- - - - Can be performed directly at the bedside, without requiring direct motor participation or speech understanding by the patient
- Allows for better differentiation between unresponsive wakefulness syndrome and minimally consciousness state
- Could be used as a tool for prognosis.

Limitations of this method:

- It requires spontaneous nasal respiration and therefore cannot be conducted with ventilated patients
- Respiration needs to be relatively stable
- Movements can generate noise to the respiration recordings
- Olfactory loss/impairment after brain trauma is common and can influence the results.

## What are the opportunities and challenges in neurodiagnostics?

The aim of neurodiagnostics is to obtain a picture of the patient's condition that is as accurate as possible. In order to achieve this, you could check whether the responsible physician adopts the following approach:

- Performs state of the art diagnosis using the tools promoted by the latest guidelines
- Is critical, self-critical and open to results
- Takes the time needed to conduct investigation and, when appropriate, repeats assessments
- Does not expect a definitive final answer from diagnostic examinations.

The initial clinical assessment of the patient can be confirmed or refuted by the results obtained by applying neurodiagnostic methods. The combination of these approaches (that is the multimodal evaluation) is important for the planning of the appropriate therapy and for the formulation of a prognosis as reliable as possible. As each method has its limitations, the advantage of combining different methods for a multimodal assessment of the patient with a disorder of consciousness is that it increases the diagnostic accuracy and the likelihood of detecting *covert consciousness* undetected by behavioural evaluation. If one particular tool cannot be used due to specific contraindications, alternative approaches may be employed to collect diagnostic data. However, the lack of a "gold standard", a proven and best method, makes diagnosis difficult. Indeed, *there is no one right method so far which yields a definitive statement on diagnosis and prognosis.* Consciousness is not directly measurable *per se*. All available clinical scales can only detect and measure the behaviour, that is the capacity to interact with the environment, hence a proxy of consciousness. In addition, patient-specific symptoms – such as motor impairments, fluctuations in wakefulness, difficulty in producing and understanding words, or sensory deficits - can mask the existing level of consciousness and can make behavioural assessment difficult. Therefore, it must be kept in mind that *no observable proof of consciousness is not a proof of no consciousness* [1].

The resulting uncertainty in the diagnosis also influences the prognosis, decisions on rehabilitation, and classification of the patient [2].


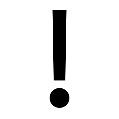
**Summary of Chapter 2 – Which recent diagnostic methods are available?**

Currently reference /standard method **Coma Recovery Scale-Revised**

- Helps to classify unresponsive wakefulness syndrome and minimally consciousness state
- No specific requirements must be met for use
- Patient’s motor participation or speech understanding are needed

Innovative methods:

**Functional magnetic resonance imaging**

- Can help to classify unresponsive wakefulness syndrome and minimally consciousness state
- Patients’ motor participation/speech understanding needed for active paradigms
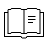

- Requirements for use: the patient must be clinically stable, must be able to be transported into the scanner, no ferromagnetic material implanted, no frequent head and body movements

**High-density electroencephalography (HD-EEG)**

- Can help to classify unresponsive wakefulness syndrome and minimally consciousness state
- Patients’ motoric participation/speech understanding needed for active paradigms
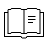

- Can be carried out in almost all patients, even in those with ferromagnetic implants

**Transcranial-magnetic-stimulation-electroencephalography (TMS-EEG)**

- Can help to classify unresponsive wakefulness syndrome and minimally consciousness state
- Requirements for use: No ferromagnetic implants near the head. No bone flap of the skull must have been removed
- Patients’ motor participation or speech understanding are not needed

**Positron-Emission-Tomography (PET)**

- Can help to classify unresponsive wakefulness syndrome and minimally consciousness state
- Requirements for use: a radioactive substance (“tracer”) must be used
- Patients’ motor participation or speech understanding are not needed

**Nasal airflow measurement**

- Can help to classify unresponsive wakefulness syndrome and minimally consciousness state
- Requirements for use: spontaneous nasal respiration and therefore cannot be conducted with ventilated patients
- Patients’ motor participation or speech understanding are not needed


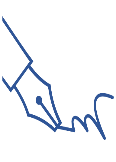


**My notes or questions on the diagnostic methods**

If you have found anything unclear and have questions while reading the chapter, you can note them down below and ask your doctor during a conversation.

Possible questions:

- What methods do you use here?
- What is the overall level of awareness of my loved one?
- Are the results of this initial assessment permanent?
  - Will this assessment be repeated? – When?
  - Is it possible that medication influenced the outcome of the assessment?
  - How will the outcome of the assessment affect further treatment and rehabilitation?

# Different behaviours in all states of disorder of consciousness

A clinical diagnosis like that of unresponsive wakefulness syndrome or minimally consciousness state distinguishes groups of patients in relation to their residual capabilities of interacting with the external environment. However, capabilities cannot only refer to the behaviour your loved one may be able to show but also to their perception of their inner or outer world (that is awareness). It is assumed that a patient’s potential to benefit from neurorehabilitation varies according to their residual capabilities. Nonetheless, within such diagnostic categories, patients can have fluctuating capabilities. These can also vary during the day, or from one day or one week to next.

- **Coma:** the eyes of the patient in coma are closed. They cannot intentionally move their body; however they can show reflexive involuntary responses to painful stimuli, to sounds, or to the touch of the skin.
- **Unresponsive wakefulness syndrome:** the eyes of the patient in unresponsive wakefulness syndrome are sometimes open and sometimes closed, indicating a partial recovery of sleep-wake cycles. The patient can show limited stereotyped reflexive reactions to external stimuli, e.g., touch or sound. However, they are not able to follow an object with their eyes, follow simple commands, or have any other meaningful interaction. It should be noted that, in rare cases, the patient cannot open their eyes due to a direct nerve damage due to their brain injury. In this case, closed eyes are not a sign of unresponsive wakefulness syndrome.
- **Minimally consciousness state:** Minimally consciousness state is a broad clinical category. In some cases, the patient with minimally consciousness state can follow people/objects with their eyes, reach for objects and manipulate them. In other cases, the patient can even be able to respond to simple commands. But the patient cannot communicate consistently at the verbal (with words) or non-verbal (without words but with facial expression, eye contact and gestures) levels.

*A patient has emerged from minimally consciousness state, when s/he is able to consistently communicate in a meaningful way, verbally or non-verbally, or when s/he is able to functionally use objects*. This can be measured with the help of the Coma Recovery Scale-Revised.

Residual capabilities of patients with disorders of consciousness are very subjective and may fluctuate, depending on the time of the day, from day to day or from week to week. The focus of this brochure is to inform you about diagnostic methods regarding disorders of consciousness. It does not aim to provide detailed information about how your loved one might feel at the moment, or what they can or cannot perceive. If you want to know more about this topic, you can consult the list below with typical questions to help you to reflect and think about the situation. These questions can be used as a basis for further discussions with doctors and relatives. Always remember that there is not one clear answer to these questions. The conversation is also influenced by the doctor's attitude or yours and whether or not he or you trust the measurements.

.

**Possible questions to start talking about your loved one with a disorder of consciousness could be:**

- Can they feel touch?
- Can they smell odours?
- Can they taste flavours of food/drink?
- Do they experience hunger/thirst?
- Can they recognise their own name?
- Can they distinguish between familiar and unfamiliar people?
- Can they remember experiences?
- Do they have dreams?
- Can they understand what others say?
- How can we support them to communicate?
- Can they store new information in memory?
- Are they aware of themselves?
- Are they capable of feeling pain?

Add your own questions:

# Disorders of consciousness and the assessment results – what does it mean for my loved one in the future?

In this brochure we present the current state of knowledge, but several ongoing studies are being conducted about diagnosis and prognosis of disorders of consciousness. Possible new findings will be incorporated in future versions of the brochure.

## What is the relevance of diagnostic test results for prognosis?

Early accurate clinical diagnosis of unresponsive wakefulness syndrome or minimally consciousness state can give useful information about the chances of long-term survival and the chances of recovering consciousness. Studies show that among patients with a prolonged disorder of consciousness, those diagnosed with minimally consciousness state within the first 5 months of injury have a more favourable long-term prognosis for functional recovery than those diagnosed with unresponsive wakefulness syndrome [3]. In addition, the early Coma Recovery Scale-Revised score can give useful information, as higher scores are associated with long-term improved responsiveness [4,5]. More detailed information on the prognosis (e.g., what other factors have an influence on recovery potential) can be provided by doctors.
Always bear in mind that individual outcomes vary from patient to patient. It is also entirely possible that a reassessment of your loved one's condition may be deemed appropriate at a later date and may produce different results.

## When does a disorder of consciousness become chronic?

Unfortunately, not every patient experiences improvement in their level of consciousness. A study from Germany has shown that up to one third of patients in inpatient rehabilitation emerges from minimal consciousness [6]. This means that in one third of the patients the condition improves. But if the condition stabilises, i.e., if there is no observable change either in one direction or the other, it is considered chronic. The following table shows the current chronological classification of disorders of consciousness [7].

| Acute (occurring quickly or suddenly) | A disorder of consciousness present for less than 28 days after the brain injury |
| --- | --- |
| Subacute (intermediate stage between acute and chronic) | More than 28 days after the brain injury |
| Chronic | More than 3 months in non-traumatic and  More than 12 months in traumatic cases after the brain injury |

## What is the difference between regained consciousness and functional/motor independence?

It is important to realise that the return of consciousness is not automatically associated with functional/motor independence. This means that the patient cannot perform movement sequences as usual. This includes movement sequences such as running, jumping, hopping (gross motor skills), but also movement sequences of hand-finger coordination and facial, eye and mouth motor skills (fine motor skills). The neural connections in the brain required for this may be irreversibly injured or new connections may no longer be possible. Training (rehabilitation), especially in the acute-subacute phase, is needed to promote the recovery of speech, communication, or motor abilities.


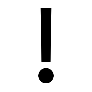
**Summary of chapter 4 – What does a disorder of consciousness mean for my loved one in the future?**

- Diagnostic category of disorders of consciousness and early Coma Recovery Scale-Revised score are important for formulating a prognosis and influence treatment options and rehabilitation planning
- Individual outcomes can vary from patient to patient
- A reassessment of your loved one´s condition may be deemed appropriate at a future date
- Return of consciousness is not automatically associated with functional/motor independence
- Ongoing rehabilitation is advised to encourage recovery and prevent secondary injuries.


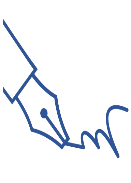


**My notes or questions in relation to the prognosis – What does the prognosis mean for me and my loved one?**

If you have found anything unclear and have questions while reading the chapter, you can note them down below and ask your doctor during a conversation.

Possible questions:

- What method and criteria did you use to make the prognosis?
- What is the prognosis for my loved one?
- What is the level of certainty of this prognosis?
- Will this prognosis be re-evaluated in the future? – When?
- What can I expect from my loved one?
- How can I best support my loved one?
- How will this prognosis affect my future and that of my loved one?

# Other topics that could be of interest

This brochure focuses on diagnostic tools to assess disorders of consciousness. Other topics that are relevant for you and your loved one may not be covered here. Therefore, we provide here a list of topics that might be of interest to you:

- What therapy options exist? Where can I find support?
- What are the rehabilitation facilities for patients with disorders of consciousness?
- Can I get psychological support? Where can I find this support?
- Can I get financial or legal support? Where can I find this support?
- What are my rights as a caregiver (e.g., a person who makes the decision for the patient in the hospital and is authorized to receive certain clinical information) Who can advise me here?

Add further topics:

In addition, on the following page specific internet addresses and aid organisations can be listed that may provide answers to your questions. This can be done in cooperation with doctors and social workers.

# Recommendations for more information

**Important addresses of non-profit associations/organisations**

**Internet sites for obtaining further information**

**Phone numbers for support should problems arise**


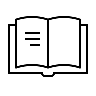
**Glossary**

**Brain tissue:** Is the networking of nerve cells that are responsible for the processing and transport of neuronal signals

**Glucose:** A sugar molecule which serves as the main source of energy for living organisms and is involved in metabolism.

**Liquor**: Is a fluid in the brain that protects it.

**Magnetic field**: Refers to the state of the space around magnets in which forces are exerted on other magnets or any other bodies, in particular, bodies made of ferromagnetic materials.

**Moustache**: Nasal cannula resembling a moustache when placed on the nose, which measures nasal respiration and breathing patterns.

**Paradigm**: Used to refer to a pattern or scheme.

**Radio waves**: Are mainly used for broadcasting (hence the name) and are caused by oscillations of electric and magnetic fields.

**Spirometer:** Device that measures the respiratory volume.

**X-ray**: A form of high energy electromagnetic radiation that can pass through most objects, including the body; with the help of an X-ray detector, the "shadows" of objects in the body are displayed.

**List of the most important abbreviations**

**DoC**: Disorder of Consciousness

**EEG**: Electroencephalography

**fMRI**: functional Magnetic Resonance Imaging

**MRI**: Magnetic Resonance Imaging

**PET**: Positron-Emission-Tomography

**TMS**: Transcranial Magnetic Stimulation**References**

[1] Fins, Joseph J. (2022). Covert Consciousness and the Right to Care: Bringing Disability Rights to the Clinic. Keynote at An Saol Foundation Summer School on Life and Living with a severe Acquired Brain Injury: Leave No One Behind. May 2022. https://www.youtube.com/watch?v=mYByrNvD0Zo

[2] Fins, Joseph J. (2015). Rights Come to Mind, Brain Injury, Ethics, and the Struggle for Consciousness. Cambridge University Press.

[3] Faugeras et al. (2018) Survival and consciousness recovery are better in the minimally conscious state than in the vegetative state, Brain Injury, 32:1, 72-77, DOI: 10.1080/02699052.2017.1364421

[4] Lucca et al. (2019). Outcome prediction in disorders of consciousness: the role of coma recovery scale revised. BMC neurology, 19(1), 68. https://doi.org/10.1186/s12883-019-1293-7

[5] Portaccio et al. (2018). Score on Coma Recovery Scale-Revised at admission predicts outcome at discharge in intensive rehabilitation after severe brain injury, Brain Injury, 32:6, 730-734, DOI: 10.1080/02699052.2018.1440420

[6] Grill et al. (2013). Rationale and Design of the Prospective German Registry of Outcome in Patients with Severe Disorders of Consciousness After Acute Brain Injury. Archives of Medicine and Rehabilitation, 94:10, 1870-1876, DOI: <https://doi.org/10.1016/j.apmr.2012.10.040>

[7] Comanducci et al. (2020). Clinical and advanced neurophysiology in the prognostic and diagnostic evaluation of disorders of consciousness: review of an IFCN-endorsed expert group. Clinical Neurophysiology 131, 2736–2765. DOI: <https://doi.org/10.1016/j.clinph.2020.07.015>

**Funding**

This brochure was developed within the “PerBrain” project, an EU-funded research project under the umbrella of the ERA PerMed Cofound. The “PerBrain” consortium involves research institutions in France, Italy, Israel, and Germany. The project work is funded by local non-profit funding organizations.
